# Host HSPD1 Translocation from Mitochondria to the Cytoplasm Induced by *Streptococcus suis* Serovar 2 Enolase Mediates Apoptosis and Loss of Blood–Brain Barrier Integrity

**DOI:** 10.3390/cells11132071

**Published:** 2022-06-29

**Authors:** Tong Wu, Li Jia, Siyu Lei, Hexiang Jiang, Jianan Liu, Na Li, Paul R. Langford, Hongtao Liu, Liancheng Lei

**Affiliations:** 1 State Key Laboratory for Zoonotic Diseases/Key Laboratory of Zoonosis, Ministry of Education, Institute of Zoonosis, College of Veterinary Medicine, Jilin University, Changchun 130062, China; twu21@mails.jlu.edu.cn (T.W.); jiali16@mails.jlu.edu.cn (L.J.); jianghx91@jlu.edu.cn (H.J.); liujn18@mails.jlu.edu.cn (J.L.); vetlina@jlu.edu.cn (N.L.); 2 School of Basic Medicine, Jilin University, Changchun 130021, China; leisy18@mails.jlu.edu.cn; 3 Section of Paediatric Infectious Disease, Imperial College London, London W2 1NY, UK; p.langford@imperial.ac.uk; 4 Department of Veterinary Medicine, College of Animal Science, Yangtze University, Jingzhou 434023, China

**Keywords:** *Streptococcus suis* serovar 2, heat shock protein family D member 1, enolase, blood–brain barrier, meningitis

## Abstract

*Streptococcus suis* serovar 2 (*S. suis* serovar 2) is a zoonotic pathogen that causes meningitis in pigs and humans, and is a serious threat to the swine industry and public health. Understanding the mechanism(s) by which *S. suis* serovar 2 penetrates the blood–brain barrier (BBB) is crucial to elucidating the pathogenesis of meningitis. In a previous study, we found that expression of the virulence factor enolase (Eno) by *S. suis* serovar 2 promotes the expression of host heat shock protein family D member 1 (HSPD1) in brain tissue, which leads to the apoptosis of porcine brain microvascular endothelial cells (PBMECs) and increased BBB permeability, which in turn promotes bacterial translocation across the BBB. However, the mechanism by which HSPD1 mediates Eno-induced apoptosis remains unclear. In this study, we demonstrate that Eno promotes the translocation of HSPD1 from mitochondria to the cytoplasm, where HSPD1 binds to β-actin (ACTB), the translocated HSPD1, and its interaction with ACTB led to adverse changes in cell morphology and promoted the expression of apoptosis-related proteins, second mitochondria-derived activator of caspases (Smac), and cleaved caspase-3; inhibited the expression of X-linked inhibitor of apoptosis protein (XIAP); and finally promoted cell apoptosis. These results further elucidate the role of HSPD1 in the process of Eno-induced apoptosis and increased BBB permeability, increasing our understanding of the pathogenic mechanisms of meningitis, and providing a framework for novel therapeutic strategies.

## 1. Introduction

*Streptococcus suis* (*S. suis*) is a major pig pathogen that causes meningitis, arthritis, septicemia, pneumonia, and endocarditis [1]. Of the 35 serovars, *S. suis* serovar 2 is the most pathogenic and has the highest clinical isolation rate [2]. Meningitis is the most serious disease manifestation caused by *S. suis* serovar 2 infections. Even if cured, irreversible long-term sequelae in humans, such as deafness, can occur [3,4,5,6]. 

Although some bacteria can access the central nervous system (CNS) via the olfactory nerve [7], most cross the blood–brain barrier (BBB) and/or the blood–cerebrospinal fluid (CSF) barrier (BCSFB) to cause central nervous system infections. Invasion and translocation of SS across the BCSFB were reported to involve three potential steps: the invasion of porcine choroid plexus epithelial cells (PCPECs) from the basolateral side; transport within membrane-bound endocytic vacuoles to the apical side; and exocytosis via the apical membrane of the BCSFB [8]. The BBB is a dynamic interface between blood and brain tissue, which can selectively block the passage of substances [9]. By destroying the integrity of the BBB structure and enhancing its permeability, pathogens can enter brain tissue and cause disease. Cerebral microvascular endothelial cells are one of the main structural cells of the BBB, and the interaction between bacteria and endothelia has uncovered molecular pathogenetic mechanisms involved in bacterial meningitis [10].

Eno is a catalytic enzyme involved in bacterial glycolysis [11], that is also surface-exposed [12], and a virulence factor of *S. suis* serovar 2 [13]. In our previous study, we demonstrated that Eno promotes the expression of host heat shock protein family D member 1 (HSPD1); Eno-HSPD1 interaction induces apoptosis of porcine brain microvascular endothelial cells (PBMECs), resulting in impairment and increased permeability of the BBB, and promotion of *S. suis* serovar 2 penetration through the barrier [14]. However, the specific role of HSPD1 in this process remains unknown.

HSPD1 has functions in protein folding, regulates apoptosis and immunocompetence, is an important chaperone molecule in mitochondria, and has an important role in the pathogenesis of tumors and infectious diseases [15]. In addition, it has been demonstrated that HSPD1 can directly interact with some pathogens’ proteins, such as the hepatitis B virus HBx protein and HIV protein gp41 [16,17]; the former promotes apoptosis. The HSPD1′s involvement in *S. suis*-serovar-2-induced apoptosis has been described in our previous study [14], which proved that Eno binds to 40S ribosomal protein SA (RPSA) and migrates from the surface of PBMECs to the cytoplasm during infection, and finally leads to HSPD1-increased expression and apoptosis. However, the exact mechanism has not been clarified. We deduce that elevated HSPD1 may play an important role in *S. suis*-serovar-2-induced apoptosis, and the objective of our study is to illustrate the role of HSPD1 and the underlying mechanism. In this study, the role of HSPD1 in the process of Eno-induced apoptosis was identified. With Eno stimulation, HSPD1 migrates from mitochondria to the cytoplasm and binds β-actin (ACTB), thereby introducing cell apoptosis via the Smac-XIAP-Caspase-3 pathway and ultimately leading to enhanced BBB permeability. This study provides a greater understanding of the pathogenic role of HSPD1 in *S. suis*-serovar-2-induced meningitis and forms the basis for new therapeutic strategies to treat bacterial meningitis.

## 2. Materials and Methods

### 2.1. Ethics Statement

All animal experimental procedures were performed in strict accordance with the Guidelines for the ethical review of laboratory animal welfare People’s Republic of China National Standard GB/T 35892 (Number of permits: TAEEI2017086).

### 2.2. Bacterial Strains, Culture Conditions, and Plasmids

*S. suis* serovar 2 strain CVCC606 (a porcine meningitis isolate) [18] was purchased from the China Veterinary Culture Collection Center, and cultured in brain heart infusion (BHI) medium (Becton, Dickinson and Company, Franklin Lakes, NJ, USA) with 5% newborn bovine serum (Clark Bioscience, Richmond, VA, USA) at 37 °C; all *Escherichia coli* strains were grown on Luria–Bertani (LB) broth agar plates and incubated for 10 h at 37 °C. Single colonies were transferred into 5 mL of LB broth and incubated for 8 h at 37 °C with agitation. pET28a:Eno prokaryotic expression plasmids have been described previously [13]. *HSPD1* and *ACTB* genes were cloned into eukaryotic expression plasmids pCMV-3×FLAG and pEGFP, respectively; for overexpressing HSPD1/ACTB in a eukaryotic cell, the primers used are shown in Table S1 (*HSPD1-F/HSPD1-R*; *ACTB-F/ACTB-R*). The constructed plasmids were named as pCMV-3×FLAG-HSPD1 and pEGFP-ACTB.

### 2.3. Recombinant Proteins HSPD1 and Eno

Plasmid pET28a expressing Eno has been described previously [13]. The coding region of the *HSPD1* gene (NCBI Reference Sequence: NM_001254716.1) was amplified from swine genomic DNA by PCR. Primers (*pET28a-HSPD1-F/pET28a-HSPD1-R*) used for amplification of *HSPD1* are listed in Table S1. The ends of the amplicon were modified with *Bam*HI and *Nd*eI, and the fragment was then ligated into the pET28a vector, which had been cut with *Bam*HI and *Nd*eI endonuclease. The plasmid was amplified in *E. coli* DH5α cells, and the plasmid was extracted with the TIANprep Mini Plasmid Kit (TIANGEN, Beijing, China). DNA sequencing of the plasmid (Sangon Biotech company, Shanghai, China) confirmed the correct insertion of *HSPD1*. 

Recombinant HSPD1 and Eno protein were expressed in *E. coli* BL21(DE3) from plasmids pET28a:HSPD1 and pET28a:Eno and purified by High Affinity Ni-NTA Resin (GenScript, Nanjing, China). Endotoxins were removed from the recombinant proteins using the Endotoxin Removal Kit (Genscript, Nanjing, China) as per the manufacturer’s instructions. Purification and determination of concentration were performed as we have described previously [13]. The protein concentration was measured with the PierceTM BCA Protein Assay Kit (Thermo, New York, NY, USA). The pure proteins were stored at −80 °C in glycerin (20%) until required.

### 2.4. Cell Cultures and Infection Experiments In Vitro

PBMECs were obtained as described previously [19] and were maintained in RPMI 1640 medium (BioInd, Beit Haemek, Israel) supplemented with 10% fetal bovine serum (Clark Bioscience, Richmond, VA, USA). Human embryonic kidney HEK-293T (293T) cells were maintained in DMEM/F12 medium (Gibco, New York, NY, USA). All cells were cultured under humidified conditions at 37 °C and 5% CO_2_.

For cell infection experiments, *S. suis* serovar 2 was cultured to logarithmic log phase (OD_600nm_ = 0.4–0.6), then centrifuged at 5000 g for 5 min, resuspended, and washed three times with sterile phosphate-buffered saline [PBS (8 g NaCl, 0.2 g KCl, 3.58 g Na_2_HPO_4_·12H_2_O and 0.27 g KH_2_PO_4_ dissolved in 1 L ddH_2_O)]. Prewashed *S. suis* serovar 2 suspensions were added to cells at a multiplicity of infection (MOI) of 50:1. Purified recombinant Eno or HSPD1 was added to PBMECs or 293T cells at a concentration of 20 µg/mL, and subsequent experiments were performed after stimulation for the indicated time.

### 2.5. Annexin V-FITC/PI Staining for Apoptosis Detection

The treated cells were collected and washed twice with ice-cold PBS, then resuspended in Annexin V binding buffer (l×) to l × 10^6^ cells/mL, and analyzed by the Annexin V-FITC Apoptosis Detection Kit (Sigma, Shanghai, China) as per the manufacturer’s instructions. The stained cells were analyzed by flow cytometry within 1 h.

### 2.6. Analysis of Expression and Distribution of HSPD1 Treated with Eno

293T cells were treated with 20 μg/mL Eno for 0 h, 6 h, 12 h, and 24 h, and the cytoplasmic and mitochondrial protein fractions of each treatment group were collected by the use of the Minute^TM^ Mitochondrial Isolation Kit (Invent Biotechnologies, Inc., Eden Prairie, MN, USA) as per the manufacturer’s instructions. Cytoplasmic and mitochondrial HSPD1 content was analyzed by Western blotting using rabbit anti-HSPD1 (Proteintech, Rosemont, IL, USA) and HRP-conjugated goat anti-rabbit IgG (ABclonal, Wuhan, China) as the primary and secondary antibodies, respectively.

293T cells were treated with recombinant Eno for 0 h, 12 h, and 24 h. Immunofluorescence staining was performed as we have described previously [20]. In brief, the treated cells were fixed with 4% paraformaldehyde, then incubated in 0.02% Triton X-100 for 20 min; after being stabilized in 10% goat serum (Solarbio, Beijing, China) at room temperature for 30 min, cells were incubated with a 1:50 dilution of rabbit anti-HSPD1 (Proteintech, Rosemont, IL, USA) at 4 °C for 16 h, then incubated with a 1:100 dilution of FITC goat anti-rabbit IgG (Bioss, Beijing, China) at room temperature for 30 min. Finally, cells were stained with 200 nM Mito-Tracker Red CMXRos (Beyotime, Shanghai, China), a mitochondrial dye, for 1 h, and the cell-permeant nuclear counterstain Hoechst 33342 (1:1000 dilution) for 5 min. Analyses were performed by confocal microscopy (Institute of Zoonosis, Jilin University). 

### 2.7. Detection of Mitochondrial Membrane Potential Marker JC-1

Cultured 293T cells were treated by Eno for 0 h, 6 h, 12 h, and 24 h. JC-1 expression was measured with the mitochondrial membrane potential assay kit as per the manufacturer’s instructions (Solarbio, Beijing, China). The samples were observed with a fluorescent microscope.

### 2.8. Expression of Genes Encoding Permeability Transformation Channel Proteins

Cultured 293T cells were treated by Eno for 0 h, 6 h, 12 h, and 24 h, and the RNA of each treatment group was extracted as follows: The cell culture medium of each treatment group was discarded, and cells gently washed with PBS. After discarding the wash fluid, 1 mL RNAiso Plus (TAKARA, Shiga, Japan) was added to the cells and the suspension pipetted up and down repeatedly. After a 5 min incubation on ice, 200 μL chloroform was added to the cell lysate and, after shaking, the mixture was placed on ice for a further 5 min, and subsequently centrifuged at 12,000 g for 15 min. The supernatant was then mixed with an equal volume of isopropanol (analytically pure) and placed on ice for 10 min. The mixture was recentrifuged, the supernatant discarded, and the precipitate resuspended in 1 mL 75% ethanol. After recentrifugation at 12,000 g at 4 °C for 5 min, the precipitate was air-dried, dissolved in 20 μL RNase-free water, and stored at −80 °C.

Fluorescence quantitative PCR (qPCR) was used to measure the expression of specific genes. RNA (800 ng) was reverse-transcribed using the PrimeScript™ RT Reagent Kit (TAKARA, Shiga, Japan) as per the manufacturer’s instructions to obtain the cDNA of the specific genes. The reaction mixture contained a final volume of 20 μL with 10 μL SYBR Green (TaKaRa, Shiga, Japan), 7.4 μL RNase-free water, 0.8 μL of each primer (10 μM), and 1 μL diluted cDNA (about 1000 ng/μL), and the expression levels of *PBR*, *Bcl-2*, *Bax*, and *GAPDH* were determined. The cycling conditions were 95 °C for 10 min and 40 cycles at 95 °C for 15 s, 60 °C for 35 s, and 60 °C for 30 s. *GAPDH* was selected as the housekeeping gene, the expression of which served as an internal reference. The relative mRNA expression level of each gene was calculated according to the 2^(−ΔΔC_T_) method. Sequences for specific primers were designed by Sangon Biotech Company (Shanghai, China) and are listed in Table S1 (*PBR-F/PBR-R*; *Bcl-2-F/Bcl-2-R*; *Bax-F/Bax-R*).

### 2.9. Cell Transfection

Cell transfection was performed as we have described previously [20]. Transfection of the DNA plasmids was performed with X-tremeGENE HP DNA Transfection Reagent (Roche, Basel, Switzerland) according to the manufacturer’s instructions. pEGFP-ACTB and pCMV-3×FLAG-HSPD1 were transfected to 293T cells, and the cell samples were used for co-immunoprecipitation (Co-IP) experiments after 24 h.

### 2.10. Co-Immunoprecipitation (Co-IP) of HSPD1 and ACTB

Co-IP was performed as we have described previously [20]. The Pierce^TM^ Classic Magnetic Bead Immunoprecipitation Kit (Thermo Fisher, New York, NY, USA) was used for immunoprecipitation experiments. The fundamental steps were followed as recommended by the manufacturer. The IP antibodies were mouse anti-GFP IgG and mouse anti-FLAG IgG. Finally, the samples were analyzed by Western blotting using mouse anti-FLAG IgG (Proteintech, Rosemont, IL, USA) or mouse anti-GFP IgG (Proteintech, Rosemont, IL, USA) as the primary antibody.

### 2.11. HSPD1 Knockdown Using siRNA

293T cells were transfected with HSPD1-siRNA (Table S2) using the X-tremeGENE siRNA Transfection Reagent (Roche, Basel, Switzerland) according to the manufacturer’s instructions. The cell pellets were collected after 24 h and lysed with RIPA lysis buffer. The lysate was collected by centrifugation at 12,000 g for 10 min, and the expression of the HSPD1 protein was analyzed by Western blotting to determine whether the target protein was successfully knocked down.

### 2.12. Western Blotting

Equal amounts of proteins were solubilized in sodium dodecyl sulfate (SDS) sample buffer, separated by SDS-PAGE, and transferred to a polyvinylidene fluoride (PVDF) membrane (Merck Millipore, Burlington, VT, USA). The membrane was incubated overnight at 4 °C with the corresponding primary antibodies. After the membranes were incubated with an HRP-conjugated secondary antibody (ABclonal, Wuhan, China) at room temperature for 1 h, the signals were detected by the ECL chemiluminescent imaging system (Tanon, Shanghai, China). Finally, ImageJ 1.53b (https://imagej.nih.gov/ij/, accessed on 5 December 2018, National Institutes of Health, Rockville, MD, USA) was used to quantify bands. All primary antibodies used in this study are listed in Table S3.

### 2.13. Establishment and Assessment of the In Vitro BBB Model

Healthy 1-month-old Landrace piglets were purchased from Jilin University Breeding pig farm, porcine PBMECs isolated, and the BBB model constructed as described previously [21]. The stability of the BBB model was assessed by measurement of transendothelial cell electric resistance (TEER), using the Millicell-ERS voltage resistance meter (Merck Millipore, Burlington, VT, USA) as per the manufacturer’s instructions. Each cell culture well was measured three times and averaged, and reported as Ω·cm^2^ after correcting for the surface area of the membrane. TEER values of PBMEC cultures were monitored from day 2 to day 8 of the experiments.

The integrity of the BBB model was evaluated by TEER as described previously [21]. HSPD1 (20 μg/mL) was added to the upper chamber of the BBB model, and PBS alone was used as the control group. The TEER values were determined at 0 h, 1 h, 2 h, 3 h, 6 h, 9 h, 12 h, 18 h, and 24 h, using the Millicell-ERS voltage resistance meter (Merck Millipore, Burlington, VT, USA) as per the manufacturer’s instructions. The TEER of each cell culture well was measured three times and averaged, and reported as Ω·cm^2^ after correcting for the surface area of the membrane.

### 2.14. Assessment of the Permeability of BBB Using Evans Blue (EB) Dye

Healthy 4- to 6-week-old female ICR mice (20–22 g) were purchased from Changchun Yisi Experimental Animal Co. Ltd. and randomly divided into 2 groups (n = 5 for each group). The animals were maintained on a 12 h light/dark cycle with free access to food and water. All animal experimental procedures were performed in strict accordance with the guidelines for the ethical review of laboratory animal welfare People’s Republic of China National Standard GB/T 35892 (Number of Permit: TAEEI2017086).

For the HSPD1 group, mice were injected with 200 μL HSPD1 (2 mg/mL) via the tail vein, and 200 μL 1% EB solution intravenously injected after 23 h. For the control group, mice were injected with 200 μL PBS via the tail vein, and 200 μL 1% EB solution intravenously injected after 23 h. Brain tissues were collected from each group at 24 h. The amount of EB in brains was determined by extraction with formamide as previously described [22]. The brain tissues were soaked in formamide and extracted at 50 °C shielded from light, and the absorbance of the supernatant at 630 nm was measured by an enzyme-linked immune detector (Becton, Dickinson and Company, Franklin Lakes, NJ, USA). The EB content in brain tissue was calculated, with results expressed as EB content per gram of brain tissue.

### 2.15. Immunohistochemical Analysis of Brain Tissues

In this study, the mouse brain tissues used for immunohistochemical analysis were produced in our earlier work [14]. Immunohistochemical analysis was performed with Histostain™-SP Kits (ZSbio, Beijing, China) according to the manufacturer’s instructions. The primary antibody used in this assay was rabbit anti-HSPD1 (1:20 dilution, Proteintech, Rosemont, IL, USA) diluted in PBS containing 1% BSA. Finally, the area around the hippocampus was observed and photographed.

### 2.16. Statistical Data Analysis

All statistical analyses were performed with GraphPad Prism 5 software (https://www.graphpad.com/, accessed on 22 December 2018, GraphPad Prism Software Inc., San Diego, CA, USA). Student’s *t*-test (2 groups), and one-way ANOVA (≥3 groups) were used to assess the statistical difference. Unless otherwise specified, data are expressed as the mean ± SD of three independent experiments. Statistically significant differences were considered as *p* values < 0.05 (*, *p* < 0.05; **, *p* < 0.01; ***, *p* < 0.001).

## 3. Results

### 3.1. HSPD1 Enhances BBB Permeability

**Figure 1:**
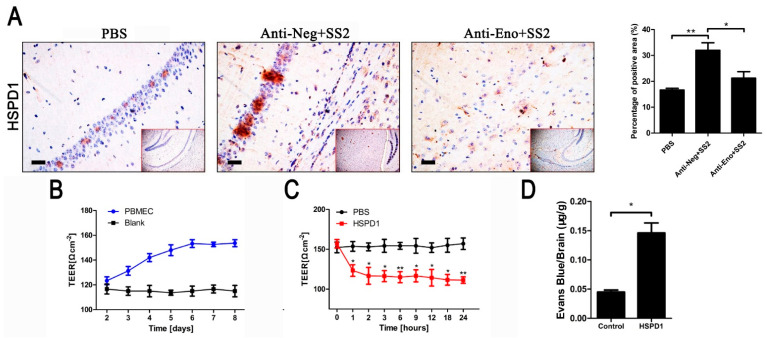
*S. suis* serovar 2 infection promotes the expression of HSPD1 which disrupts BBB in vivo and in vitro. (**A**) Immunohistochemical analysis of the mouse brain tissues from PBS, Anti-Neg+SS2, and Anti-Eno+SS2 groups (scale bar, 100 μm). (**B**) BBB model’s TEER changes. The Blank group equals the empty trans-well without cells. (**C**) BBB model’s TEER changes with or without HSPD1 treatment. (**D**) EB content of mouse brain tissues after HSPD1 treatment or in the untreated control group. (*S. suis* serovar 2, SS2: *Streptococcus suis* serovar 2; HSPD1: heat shock protein family D member 1; BBB: blood–brain barrier; PBS: phosphate-buffered saline; TEER: trans-endothelial cell electric resistance; EB: Evans blue.) (*, *p* < 0.05; **, *p* < 0.01).

Eno-RPSA interaction promotes HSPD1 expression, which in turn promotes PBMECs apoptosis [14]. To determine if *S. suis* serovar 2 induces increased expression of HSPD1 in mouse brain tissue, ICR mice were injected intraperitoneally with *S. suis* serovar 2. The brains were collected at 24 h and immunohistochemical results (Figure 1A) showed that the level of HSPD1 in brain tissue was increased after *S. suis* serovar 2 infection (the brown and yellow stained area indicates HSPD1 expression). When the Eno antibody was injected before *S. suis* serovar 2 infection, the expression of HSPD1 was significantly reduced compared with the anti-Neg group (Figure 1A). These results demonstrate that the expression of HSPD1 in the brain tissue was increased with *S. suis* serovar 2 infection and this, to some extent, was associated with Eno. 

Next, the effect of recombinant HSPD1 protein on BBB permeability, as measured by TEER, was analyzed in the in vitro BBB model previously described [21]. As in our previous work [14], the TEER value of PBMECs gradually increased from day 2 to 6, and thereafter stabilized, indicating that PBMECs formed a dense monolayer barrier with low permeability and good barrier function (Figure 1B). Compared with the PBS group, the TEER value of the recombinant-purified HSPD1 (Figure S5) treatment group was significantly decreased (Figure 1C), which indicated that the BBB model was damaged and permeability increased. EB is a dye that cannot enter brain tissue if the BBB is intact [22]. We, therefore, used EB to determine the intactness of BBB in vivo. The results showed that the injection of HSPD1 via the tail vein was associated with a significant increase in the EB content in mouse brain tissue (Figure 1D). These results verified that *S. suis* serovar 2 induction or the presence of HSPD1 increases the permeability of the BBB both in vitro and in vivo.

### 3.2. Increased HSPD1 Promotes PBMECs and 293T Cells Apoptosis

**Figure 2:**
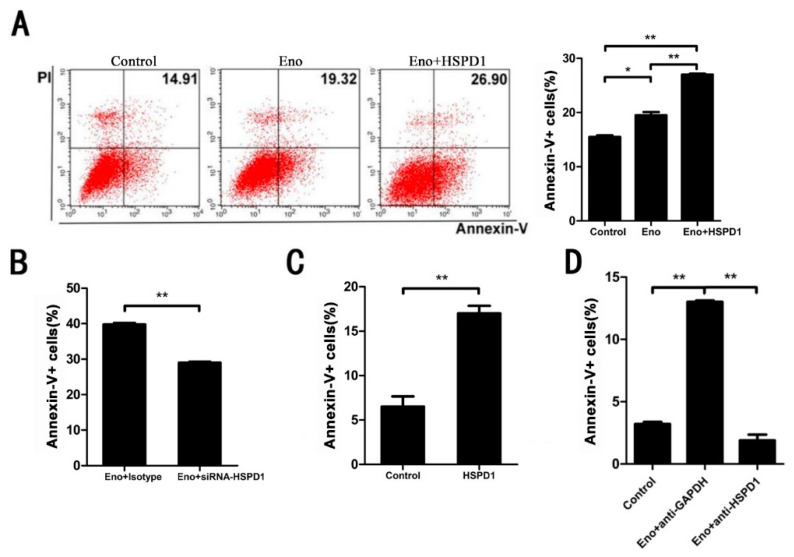
Both intracellular and extracellular HSPD1 can lead to apoptosis. Flow cytometry analysis was performed to detect Eno- or HSPD1-induced apoptosis. (**A**) HSPD1 was overexpressed in 293T cells by transfection of eukaryotic expression plasmid pCMV-3×FLAG-HSPD1, and its effect on Eno-induced apoptosis was analyzed. (**B**) HSPD1 was knocked down in 293T cells by transfection of HSPD1-siRNA, and its effect on Eno-induced apoptosis was analyzed. (**C**) PBMECs were treated with purified HSPD1 protein extracellularly, and the level of apoptosis was determined. (**D**) Anti-HSPD1 IgG prevented extracellular-HSPD1-induced PBMEC apoptosis. The anti-GAPDH group was used as the isotype control. (The numbers in the scatter diagrams represent the percentage of Annexin-V positive cells; the gating strategies are shown in Figure S2; the scatter diagrams of (**B**–**D**) are shown in Figure S3) (PBMECs: porcine brain microvascular endothelial cells) (*, *p* < 0.05; **, *p* < 0.01).

To determine whether HSPD1 directly affects apoptosis which has been induced by Eno-RPSA interaction, we overexpressed HSPD1 in the cytoplasm of 293T cells and found that Eno-induced apoptosis was significantly promoted, compared with Eno treatment alone (Figure 2A). Conversely, when we knocked down HSPD1 by siRNA, apoptosis was significantly reduced (Figure 2B), which indicated that HSPD1 is correlated with apoptosis. Additionally, adding purified HSPD1 to PBMECs resulted in significant apoptosis, and Eno-induced apoptosis could be blocked by anti-HSPD1 antibody (Figure 2C,D), which suggested the increase in intracellular and extracellular HSPD1 led to apoptosis. It has been previously demonstrated that extracellular HSPD1 induces apoptosis through Toll-like receptor 4 (TLR4) [23,24]. Indeed, extracellular blocking of TLR4 by rabbit anti-TLR4 IgG (Proteintech, Rosemont, IL, USA) reduced HSPD1-induced apoptosis (Figure S1). Taken together, the results suggested that HSPD1 is directly related to Eno-induced apoptosis, and increased expression of HSPD1 promotes cell apoptosis.

### 3.3. HSPD1 Translocates from Mitochondria into the Cytoplasm after Eno Treatment

**Figure 3:**
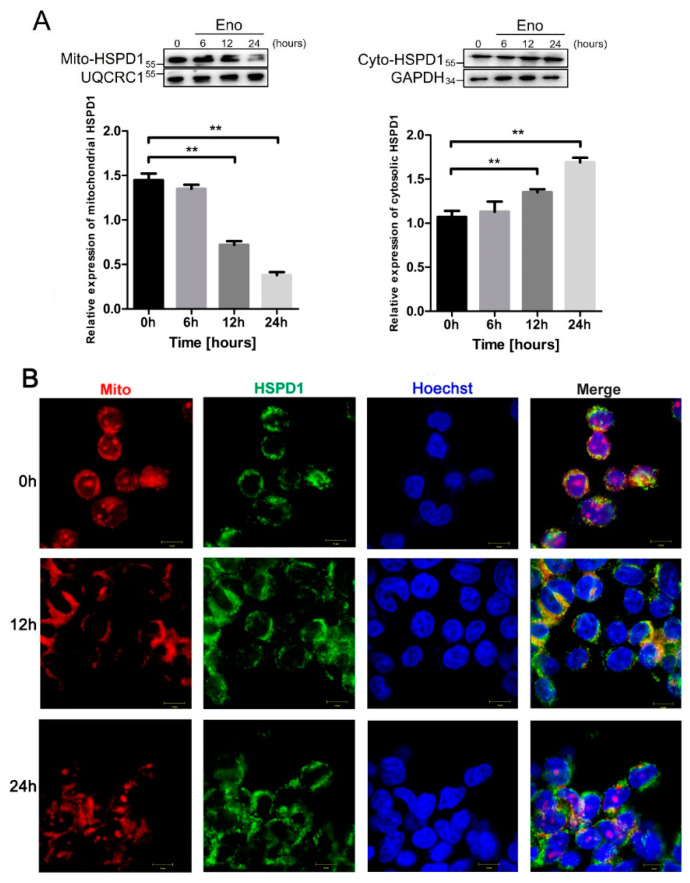
HSPD1 translocates from mitochondria into the cytoplasm after Eno treatment. 293T cells were treated with Eno and incubated for 0 h, 6 h, 12 h, and 24 h at 37 °C. (**A**) Western blotting was used to determine the expression of HSPD1 in protein fractions derived from mitochondrial and cytoplasmic compartments at different time points after Eno treatment. UQCRQ1 and GAPDH were selected as the housekeeping proteins. (**B**) Immunofluorescence to determine the localization of HSPD1 and mitochondria at different time points after Eno treatment. (Scale bar, 10μm) (**, *p* < 0.01).

HSPD1 mainly exists in mitochondria and may be transferred under stress conditions to the cytoplasm, so we hypothesized that the translocation of HSPD1 would occur after Eno treatment. Therefore, we determined the amount of HSPD1 in protein fractions derived from mitochondrial and cytoplasmic compartments at different time points after Eno treatment. The results showed that there was a time-dependent Eno-induced increase in expression of HSPD1 in the cytoplasm, while mitochondrial HSPD1 expression gradually decreased (Figure 3A). Immunofluorescence (IF) images showed that HSPD1 colocalized with mitochondria at 0 h. However, after Eno treatment, HSPD1 mitochondrial colocalization diminished with time, with gradual transfer of the protein to the cytoplasm which was near-complete at 24 h (Figure 3B). These results indicated that 293T cells stimulated by Eno induced HSPD1 to translocate from mitochondria into the cytoplasm. 

### 3.4. Eno Increases the Permeability of the Mitochondrial Membrane

**Figure 4:**
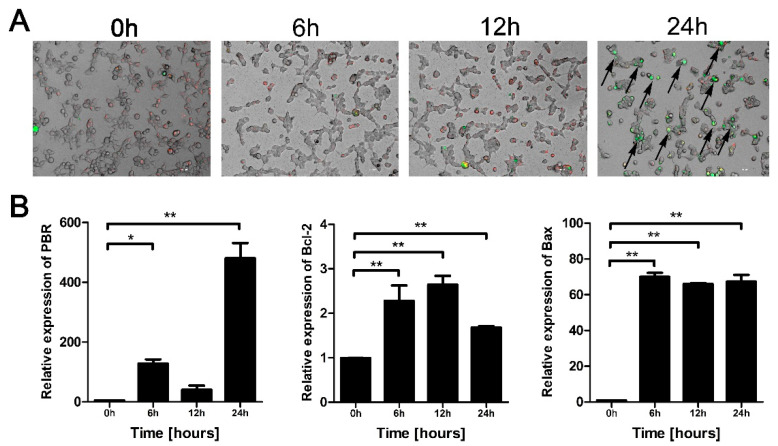
Eno induces increased permeability of the mitochondrial membrane. 293T cells were treated with Eno and incubated for 0 h, 6 h, 12 h, and 24 h; then, the mitochondrial membrane potential and expression of PTPs were analyzed. (**A**) Mitochondrial membrane potential marker JC-1 was detected. When the mitochondrial membrane potential is high, JC-1 concentrates in the mitochondrial matrix and forms a complex with Mito-Tracker Red CMXRos which produces red fluorescence. In contrast, JC-1 does not concentrate in mitochondria when the membrane potential is at a low level, represented by green fluorescence. (Scale bar, 200 μm) (**B**) qPCR to determine the relative transcriptional levels of PTP-encoding genes (including *PBR*, *Bcl-2*, and *Bax*) of Eno-treated cells. (PTPs: permeability transition pore proteins) (*, *p* < 0.05; **, *p* < 0.01).

We also hypothesized that HSPD1 transport from mitochondria to the cytoplasm is related to mitochondrial osmotic function. Therefore, we analyzed the mitochondrial membrane potential and expression of channel-related permeability transition pore proteins (PTPs, including PBR, Bcl-2, and Bax) in 293T cells [25]. Eno induced a time-dependent decrease in the mitochondrial membrane potential marker JC-1, as indicated by the black arrows in Figure 4A (a change from red to green fluorescence indicates JC-1 expression). In addition, the expression of genes encoding the mitochondrial membrane PTPs was significantly increased after Eno treatment (Figure 4B). These results indicated that the permeability of the mitochondrial membrane was increased with the opening of the PTPs, and this process assisted the translocation of HSPD1 from mitochondria to the cytoplasm, and the link with Eno-induced apoptosis.

### 3.5. ACTB Is Increased after Eno Treatment and Interacts with Translocated HSPD1 in the Cytoplasm

**Figure 5:**
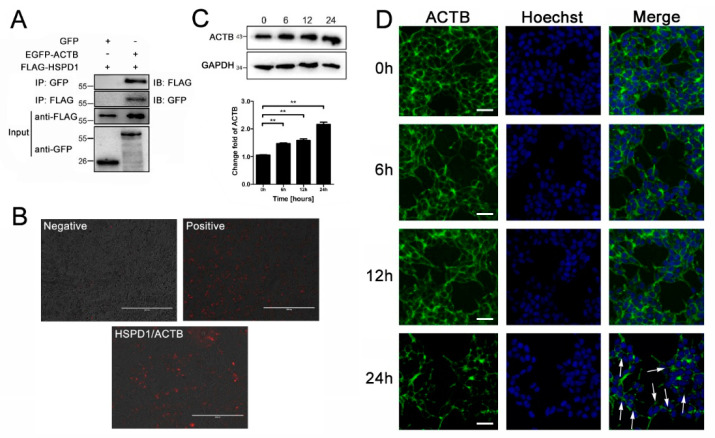
Eno promotes the expression of ACTB which interacts with HSPD1. (**A**) HSPD1-ACTB interaction shown by Co-IP. (**B**) HSPD1-ACTB interaction shown by BiFC. (Scale bar, 400 μm) (**C**) Western-blotting detection of ACTB expression in 293T cells after Eno treatment for 0, 6, 12, and 24 h. (**D**) Eno-induced morphological changes (indicated by white arrows) in cellular structures shown by immunofluorescent experiments. (Scale bar, 50 μm) (ACTB: β-actin; Co-IP: co-immunoprecipitation; BiFC: bimolecular fluorescence complementary) (**, *p* < 0.01).

We suspected that HSPD1 transfers to the cytoplasm and then interacts with other molecules to mediate apoptosis, and candidates were identified by pull-down and LC-MS/MS analysis. ACTB was identified as the protein with the strongest interaction with HSPD1 in PBMECs (Figure S4). Co-IP and bimolecular fluorescence complementation (BiFC) were performed to verify the relationship between HSPD1 and ACTB in 293T cells, and the results were consistent with the pull-down assays, i.e., HSPD1 interacts with ACTB (Figure 5A,B). Moreover, Eno treatment significantly promoted ACTB expression in 293T cells in a time-dependent manner (Figure 5C), and this increase was accompanied by changes in the cellular structure, such as gaps, irregular edges in the peripheral dense zone, and the gradual appearance of serrated structures (indicated by the white arrows in Figure 5D). These results showed that Eno promotes the expression and abnormal morphological changes in ACTB, and translocated HSPD1 interacts with ACTB in the cytoplasm; these processes may involve apoptosis.

### 3.6. HSPD1 Induces Apoptosis via the Smac-XIAP-Caspase-3 Pathway by Binding to ACTB

**Figure 6:**
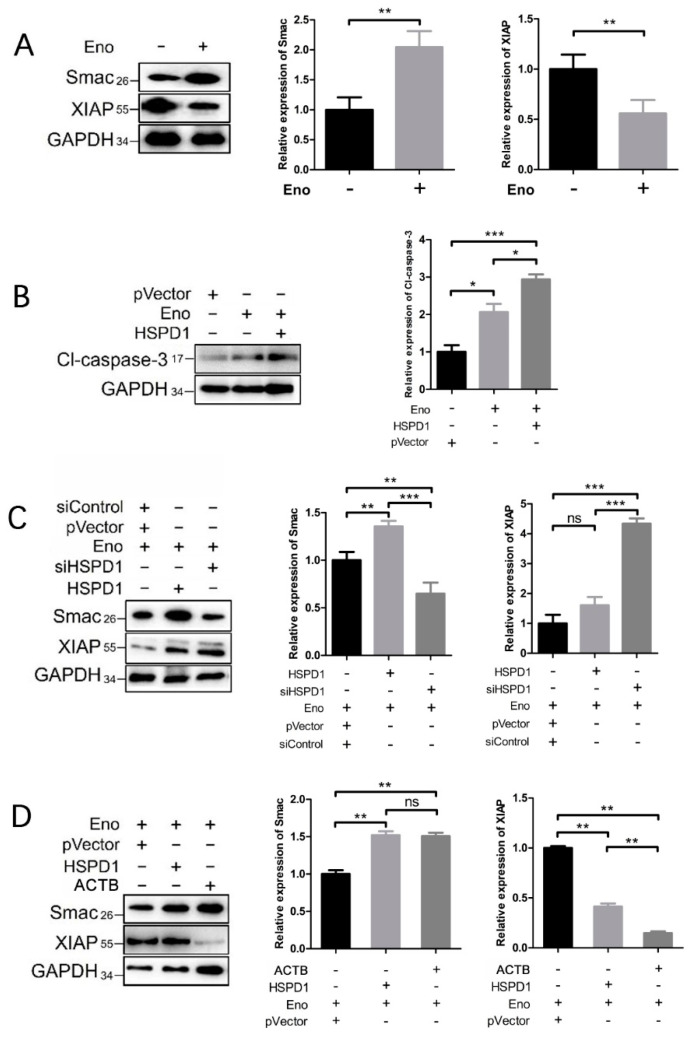
Interacting proteins HSPD1 and ACTB induce apoptosis via the Smac-XIAP-caspase-3 pathway. All Eno groups (Eno +) were treated with Eno for 24 h, and protein expression levels were determined by Western blotting. (**A**) Smac and XIAP expression levels after Eno treatment alone. (**B**) The cl-caspase-3 expression after Eno treatment alone or combined with HSPD1 overexpression. (**C**) Smac and XIAP expression levels after Eno treatment alone or combined with HSPD1 overexpression/knockdown. (**D**) Smac and XIAP expression after Eno treatment alone or combined with HSPD1/ACTB overexpression. (Eno +, 293T cells treated with Eno for 24 h; HSPD1 +, overexpression of HSPD1 in 293T cells by transfection of pCMV-3×FLAG-HSPD1; ACTB +, overexpression of ACTB in 293T cells after transfection of pEGFP-ACTB; siHSPD1 +, knockdown of HSPD1 in 293T cells by transfection of HSPD1-siRNA; pVector and siControl are the negative controls for overexpression and knockdown, respectively) (Smac: Second mitochondria-derived activator of caspases; XIAP: X-linked inhibitor of apoptosis protein; Cl-caspase-3: cleaved caspase-3) (ns, *p* ≥ 0.05; *, *p* < 0.05; **, *p* < 0.01; ***, *p* < 0.001).

**Figure 7:**
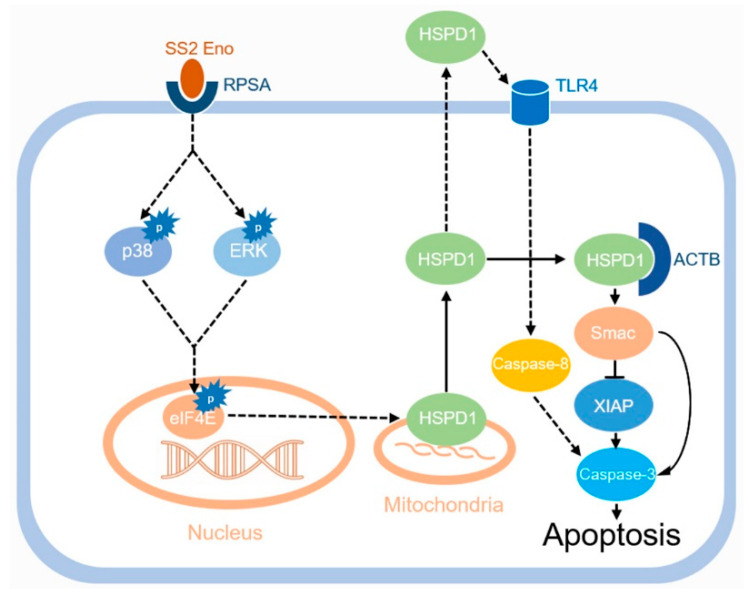
Proposed pathway of Eno-HSPD1 induced apoptosis of PBMECs. Eno promotes the translocation of HSPD1 from mitochondria into the cytoplasm, elevated HSPD1 interacts with ACTB in the cytoplasm, and this process promotes Smac expression, which in turn inhibits XIAP, and ultimately activates caspase-3, leading to apoptosis. (The pathway marked with dotted lines represents the results of our previous study).

The protein Smac (second mitochondria-derived activator of caspases) is found in mitochondria and involved in apoptosis promotion [26,27]. We, therefore, investigated the effect of Eno treatment on the expression of Smac and its related proteins, cleaved caspase-3 (Cl-caspase-3), and anti-apoptosis protein X-linked inhibitor of apoptosis protein (XIAP) in 293T cells. Eno significantly promoted the expression of Smac and Cl-caspase-3 and inhibited the expression of XIAP, which increased apoptosis (Figure 6A,B). When HSPD1 was overexpressed, the expression of Cl-caspase-3 was further enhanced (Figure 6B). Eno-induced increased Smac expression was further enhanced by HSPD1 overexpression (although the expression of XIAP did not change significantly) and could be inhibited by HSPD1-siRNA transfection, with the XIAP expression significantly increased (Figure 6C). The overexpression of ACTB also significantly enhanced the Eno-induced increase in Smac and inhibited the expression of XIAP (Figure 6D). The above results indicated that the interaction of HSPD1 and ACTB promotes the expression of Smac, which inhibits the expression of XIAP, and these processes in turn promote the cleavage of pro-caspase-3 which results in apoptosis (Figure 7).

## 4. Discussion

*S. suis* serovar 2 is most commonly isolated from human infection cases, accounting for 74.7% [2,28]. *S. suis* serovar 2 can pass through the BBB formed by brain microvascular endothelial cells (BMECs) and/or choroid plexus epithelial cells. Once bacteria enter the brain tissue, meningitis is the most serious clinical manifestation of *S. suis* serovar 2 infection, which is difficult to treat because of the difficulty of delivery of therapeutic drugs to the brain, but also the associated long-term sequelae [29]. Therefore, elucidating the mechanism of *S. suis* serovar 2 invasions to BBB provides an important theoretical basis for the study of the pathogenesis of the disease and the development of therapeutic drugs, and is of great significance for the prevention and treatment of meningitis. 

However, at present, the mechanism of *S. suis* serovar 2 crossing the BBB has not been completely concluded. In the past, many studies have focused on the important role of virulence factors of meningitis pathogens, such as type IV pili protein of *Neisseria meningitidis*, surface-anchored sialidase NanA of *Streptococcus pneumoniae*, and *S. suis* virulence factor suilysin, which have been demonstrated to play roles in inducing BMECs injury and passage across the BBB to the CNS [30,31,32,33]. On the cellular side, many important molecules that contribute to the development of meningitis have also been identified. *S. suis* serovar 2 interaction with BMECs induces serine/threonine kinase activity that affects the expression of E3 ubiquitin ligase HECTD1, which subsequently increases the degradation of claudin-5, thus enabling *S. suis* serovar 2 to traverse the BBB [34]. We had previously found that *S. suis* serovar 2 Eno, as a virulence factor involving meningitis, promotes HSPD1 expression in PBMECs by targeting RPSA, which in turn induced apoptosis, hurting the BBB integrality [14]. Here, based on our previous study, we further discovered that HSPD1 can directly bind to ACTB to enhance apoptosis through the Smac-XIAP-caspase-3 pathway, resulting in impairment of the BBB. Our results contribute to the understanding of the mechanism of *S. suis* serovar 2 invading BBB.

HSPD1, also called heat shock protein 60, is considered one of the mitochondria’s most important chaperone molecules. HSPD1 participates in protein folding and regulates apoptosis and immunocompetence, and plays an important role in tumor and infectious disease pathogenicity [15]. In this study, we showed that HSPD1 has a role in bacterial pathogen (*S. suis* serovar 2)-induced apoptosis. 

We demonstrated that increased levels of HSPD1 promote Eno-induced apoptosis. A previous study in the human Jurkat T lymphocytic cell line found that 70–80% of HSPD1 is present in mitochondria but can be mobilized by stress or other stimuli [35]. Our results show that Eno treatment is associated with the movement of HSPD1 from mitochondria to the cytoplasm, which causes cytosolic accumulation of HSPD1. HSPD1 accumulation in the cytosol after release from mitochondria has previously been interpreted as HSPD1 having a pro-death function [36]. We also found an Eno-induced time-dependent decrease in mitochondrial membrane potential marker JC-1 and an increase in the expression of genes encoding the mitochondrial membrane PTPs. We concluded that mitochondria have increased permeability in response to Eno-induced apoptosis and that molecules normally present in mitochondria, such as HSPD1, increase in concentration in the cytoplasm, and can be considered a proapoptotic effect. 

Besides HSPD1, another protein released from the mitochondria of apoptotic cells is Smac. The latter binds to apoptotic-inhibiting proteins, such as XIAP which inhibits caspases, allowing apoptosis to proceed [37]. Overexpression of HSPD1 resulted in increased Smac and decreased XIAP expression. We further identified ACTB as a protein interacting with HSPD1 in the cytosol. In 293T cells overexpressing ACTB, Smac expression was increased. Overall, these results suggested that Eno-treatment-induced HSPD1 in the cytoplasm interacts with ACTB, and increases Smac expression, which results in the activation of caspase-3 and induction of apoptosis. It has been reported that HSPD1 can form a complex with pro-caspase-3, in particular in the cytosol, and this association promotes caspase-3 maturation and activation [36]; our study confirmed ACTB mediating HSPD1-induced apoptosis, but how HSPD1-ACTB interaction influences the process is unclear.

Although the interaction between HSPD1 and pathogen proteins has been demonstrated [16,17], there is a lack of studies on its known inhibitors or regulators that could represent potential therapeutic agents in infectious diseases. At present, HSPD1 has also been identified to function in tumor progression and modulation of antitumor immune responses [38,39,40]; therefore, researchers have advanced the hypothesis that HSPD1 can be used as a target for anticancer therapy. Fortunately, HSPD1 inhibitors or regulators are confirmed to be able to modify and regulate HSPD1 expression and functions and, for this reason, their use can be switched from cancer therapy to anti-infective therapy, or treatment for meningitis. 

Our results first explored the ACTB as the binding target of HSPD1 during *S. suis* infection, and not only confirmed that inhibition of HSPD1 can effectively reduce *S. suis* serovar 2 Eno-induced apoptosis, maintain the integrity of BBB, and resist meningitis, but also provides another potential target for control infection. A problem, however, is that only a limited number of compounds have been fully characterized; for most of these inhibitors, the mechanism of action is still undisclosed. Future research should focus on revealing the mechanism of action of HSPD1/ACTB inhibitors, which have great potential for treating meningitis if they can be ensured to be safe in treated animals and humans.

## 5. Conclusions

A previous report showed that HSPD1 mediates Eno-induced PBMEC apoptosis. Eno elevates the level of HSPD1 in the cytosol through the p38-ERK-eIF4E pathway by interaction with RPSA [14]. In this study, the specific role of HSPD1 involved in Eno-induced apoptosis was revealed. We demonstrated for the first time that Eno promotes the translocation of HSPD1 from mitochondria to the cytoplasm, and discovered ACTB as the interactive protein of HSPD1 in the cytoplasm. The HSPD1-ACTB interaction in the cytoplasm causes cell morphological changes and promotes apoptosis through the Smac-XIAP-caspase-3 pathway. This network results in increased permeability of the BBB and *S. suis* serovar 2 invasion. Further investigation is required to find out how HSPD1-ACTB interaction influences the process, and the specific mechanisms involved in HSPD1-associated proapoptotic complexes.

## Data Availability

Not applicable.

## Supplementary Materials

The following supporting information can be downloaded at: https://www.mdpi.com/article/10.3390/cells11132071/s1. Figure S1. Extracellular HSPD1 induces apoptosis through TLR4. Extracellular blocking of TLR4 by anti-TLR4 antibody reduced the HSPD1-induced apoptosis. (TLR4: Toll-like receptor 4); Figure S2. Gating strategies of flow cytometry for cell apoptosis detection. A–C correspond to the flow analyses in Figure 2A, respectively; Figure S3. Scatter diagrams are used to analyze apoptosis levels. A to D correspond to A–D in Figure 2; Figure S4. Silver staining result of pull-down to identify interactive proteins of HSPD1 intracellularly. The band of interest was cut and analyzed by LC-MS/MS; Figure S5. HSPD1 protein is correctly expressed and purified. (A) The amplified fragment of HSPD1 from constructed plasmid pET28a:HSPD1 had the predicted size of 1722 bp. (B) SDS-PAGE band of purified recombinant HSPD1 was at the correct size of 63 kDa. (C) Purified HSPD1 was detected by Western blotting with mouse anti-His-Tag as the primary antibody, which recognizes the 63 kDa band; Table S1. The primers used in this study; Table S2. The siRNA used for HSPD1 knockdown; Table S3. The list of primary antibodies in this study.

## Abbreviations

SS2	*Streptococcus suis* serotype 2
BBB	Blood–brain barrier
Eno	Enolase
HSPD1	Heat shock protein family D member 1
PBMEC	Porcine brain microvascular endothelial cell
ACTB	β-actin
BCSFB	Blood–cerebrospinal fluid barrier
PCPECs	Porcine choroid plexus epithelial cell
PBS	Phosphate buffered saline
qPCR	Fluorescence quantitative PCR
Co-IP	Co-immunoprecipitation
TEER	Transendothelial cell electric resistance
EB	Evans blue
CFU	Colony-forming unit
TLR	Toll-like receptor
PTP	Permeability transition pore protein
BiFC	Bimolecular fluorescence complementary
Smac	Second mitochondria-derived activator of caspases
XIAP	X-linked inhibitor of apoptosis protein
