# Supplementary material for: Host HSPD1 Translocation from Mitochondria to the Cytoplasm Induced by Streptococcus suis Serovar 2 Enolase Mediates Apoptosis and Loss of Blood–Brain Barrier Integrity
Source: Cells. 2022 Jun 29;11(13):2071. doi: 10.3390/cells11132071 (PMC9265368; doi:10.3390/cells11132071)
Supplement: Supplementary file 1 [file cells-11-02071-s001.zip › cells-1766372-supplementary.pdf]

Table S1. The primers used in this study.

Name	Primer sequences (5'–3')
HSPD1-F	TGACGATGACAAGCTTGCGGCCGCGATGCTTCGATTACCCG-CAGTC (pCMV-3×FLAG)
HSPD1-R	GAGGGGTCACAGGGATGCCACCCGGTTAGAACATGCCAC-CTCCCATAC (pCMV-3×FLAG)
ACTB-F	CGAGCTGTACAAGTCCGGACTCAGAATGGATGACGA-TATTGCT (pEGFP)
ACTB-R	GGGCCCGCGGTACCGTCGACTGCAGCTAGAAGCATTT-GCGGTG (pEGFP)
pET28a-HSPD1-F	CGCGGATCCATGCTTCGGTTAC (*Bam*H I)
pET28a-HSPD1-R	GGGAATTCCATATGTTAGAACATGCC (*Nde* I)
PBR-F	ACAGAGAAGGCTGTGGTTCC
PBR-R	CGCCATACGCAGTAGTTGAG
Bcl-2-F	GAGGATTGTGGCCTTCTTTG
Bcl-2-R	ACAGTTCCACAAAGGCATCC
Bax-F	TTTGCTTCAGGGTTTCATCC
Bax-R	CAGTTGAAGTTGCCGTCAGA
GAPDH-F	GTCGGTTGTGGATCTGACCT
GAPDH-R	AGCTTGACGAAGTGGTCGTT

Table S2. The siRNA used for HSPD1 knockdown.

Name	Target sequences (5'–3')
HSPD1-siRNA	GCAGATGCTGTAGCTGTTA<br>GCTGTAATTGCTGAACTTA<br>CCAGCCTTGGATTCATTAA

Table S3. The list of primary antibodies in this study.

Protein name	Primary antibodies	Brand	Dilution ratio for WB
HSPD1	HSP60 Polyclonal antibody (rabbit)	Proteintech, USA	1:5000
ACTB	Actin (C-2) mouse monoclonal antibody (rabbit)	SANTA CRUZ BIO-TECHNOLOGY, USA	1:1000
GFP-tag	GFP Tag Polyclonal Antibody (rabbit)	Proteintech, USA	1：4000
FLAG-tag	DYKDDDDK tag Monoclonal antibody (mouse)	Proteintech, USA	1:2000
His-tag	6*His, His-Tag Monoclonal An-tibody (mouse)	Proteintech, USA	1:5000
Smac	DIABLO Polyclonal Antibody (rabbit)	Proteintech, USA	1:5000
XIAP	XIAP Monoclonal Antibody (mouse)	Proteintech, USA	1:5000
Cleaved caspase-3	Cleaved Caspase-3 (Asp175) (5A1E) Rabbit mAb	Cell Signaling Technol-ogy, USA	1:1000

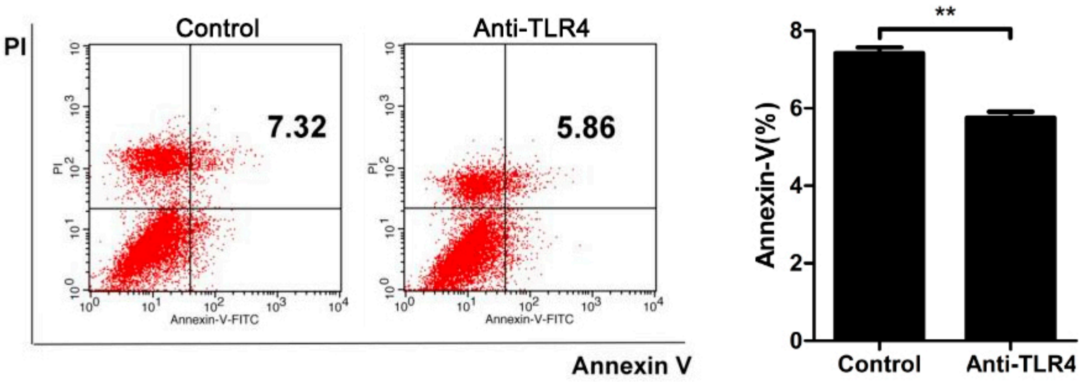

Figure S1. Extracellular HSPD1 induces apoptosis through TLR4. Extracellular blocking of TLR4 by anti-TLR4 antibody reduced the HSPD1-induced apoptosis. (TLR4: Toll-like receptor 4). (**, $p < 0.01$).

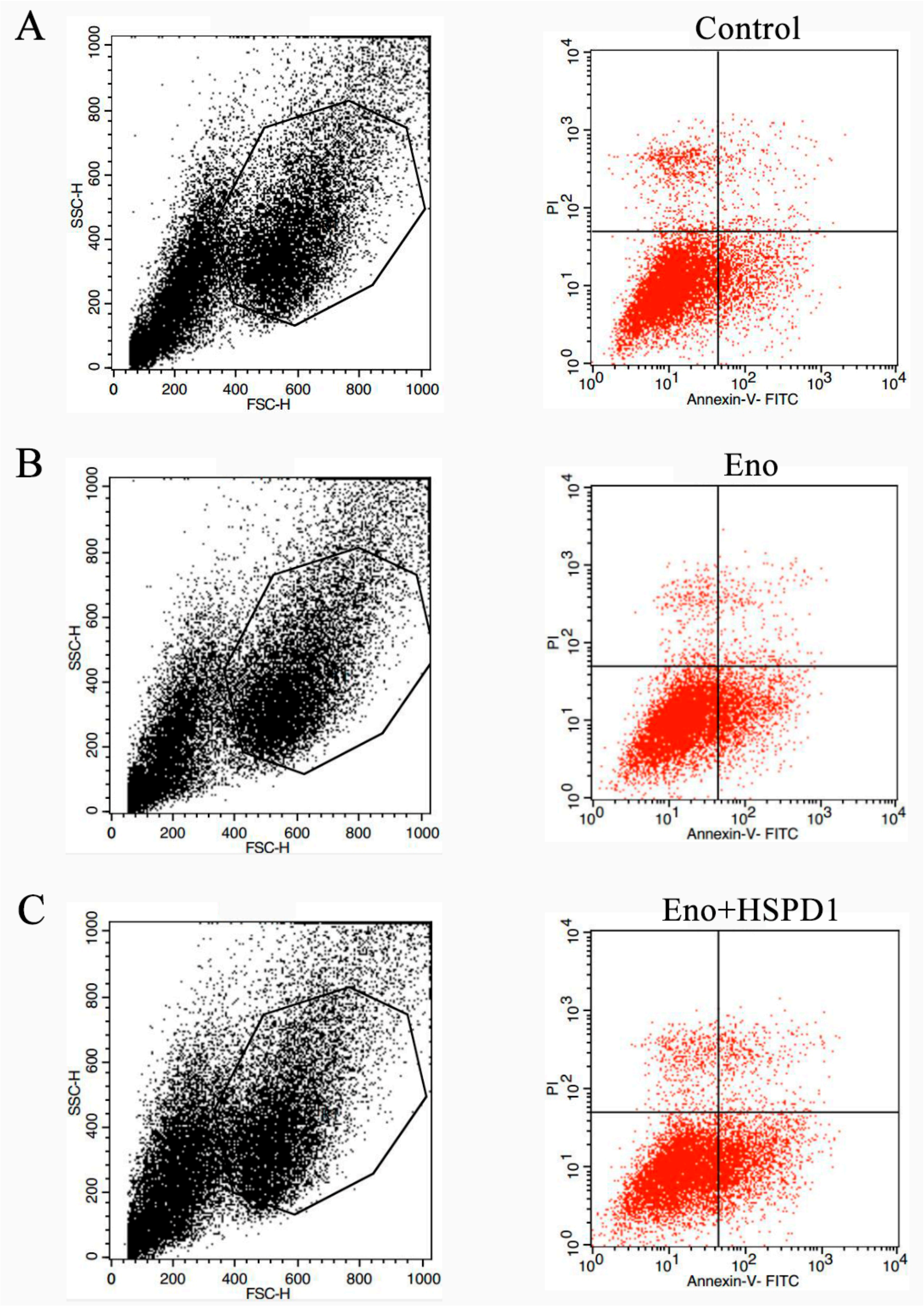

Figure S2. Gating strategies of flow cytometry for cell apoptosis detection. (**A**–**C**) correspond to the flow analyses in Figure 2A, respectively.

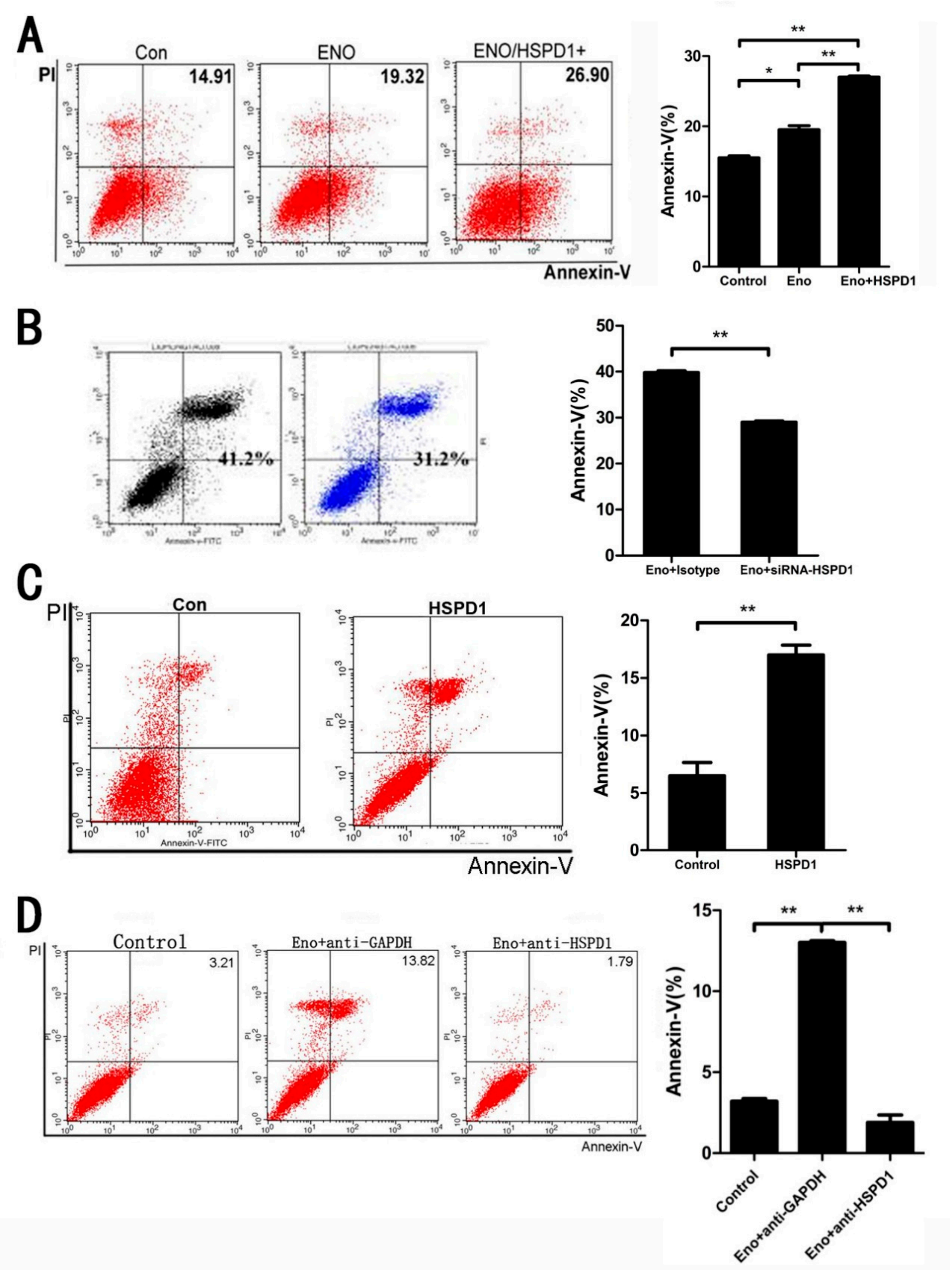

Figure S3. Scatter diagrams are used to analyze apoptosis levels. (**A**–**D**) correspond to (**A**–**D**) in Figure 2. (*, $p < 0.05$; **, $p < 0.01$).

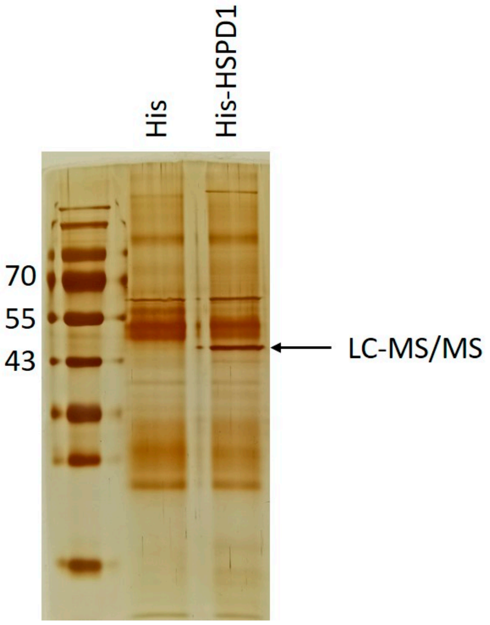

Figure S4. Silver staining result of pull-down to identify interactive proteins of HSPD1 intracellularly. The band of interest was cut and analyzed by LC-MS/MS.

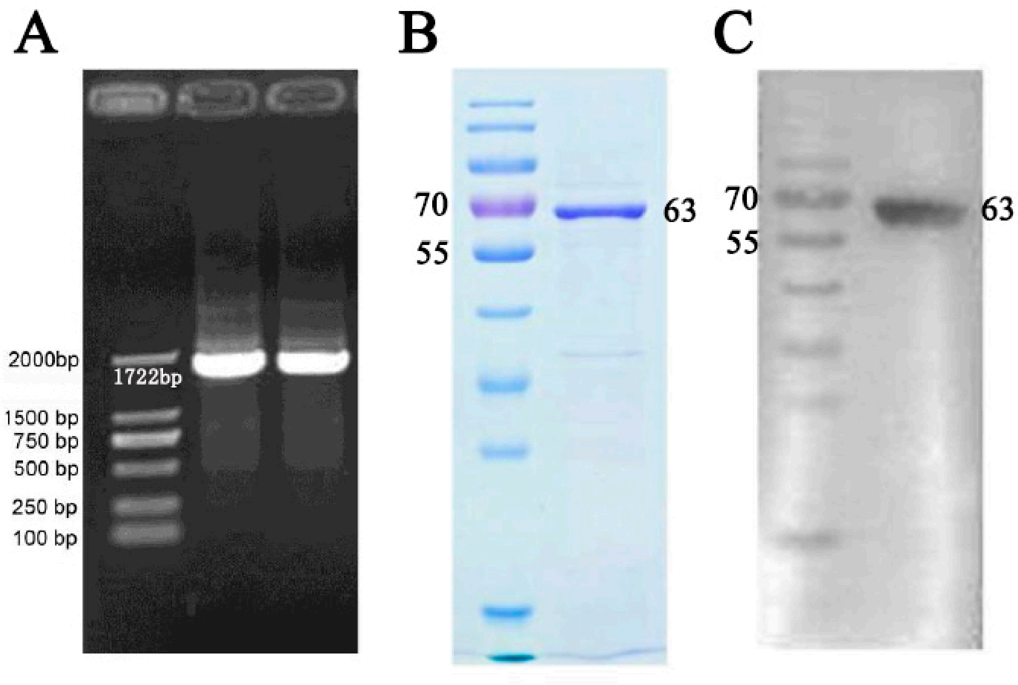

Figure S5. HSPD1 protein is correctly expressed and purified. (**A**) The amplified fragment of HSPD1 from constructed plasmid pET28a:HSPD1 had the predicted size of 1722 bp. (**B**) SDS-PAGE band of purified recombinant HSPD1 was at the correct size of 63 kDa. (**C**) Purified HSPD1 was detected by Western blotting with mouse anti-His-Tag as the primary antibody, which recognizes the 63 kDa band.
